# Prognostic value of gasping for short and long outcomes during out-of-hospital cardiac arrest: an updated systematic review and meta-analysis

**DOI:** 10.1186/s13049-018-0575-1

**Published:** 2018-12-14

**Authors:** Qiang Zhang, Bo Liu, Zhijiang Qi, Chunsheng Li

**Affiliations:** Department of Emergency Medicine, Beijing Chao-Yang Hospital,Capital Medical University, 8# Worker’s Stadium South Road, Chao-Yang District, Beijing, 100020 China

**Keywords:** Gasping, Cardiac arrest;meta

## Abstract

**Objective:**

We systematically reviewed the literature to investigate whether gasping could predict short and long outcomes in patients with out of hospital cardiac arrest (OHCA).

**Methods:**

PubMed, Embase, and Cochrane Library were searched for observational studies regarding the prognostic effect of gasping on short and long outcomes in adults with OHCA. The primary outcome was return of spontaneous circulation (ROSC). The secondary outcomes were favorable neurological outcome at discharge or at 30 days after cardiac arrest;long term (≥6 months) survival; initial shockable rhythm.The Mantel-Haenszel method with random-effects model was used to calculate pooled relative risks (RRs) and 95% confidence intervals (CIs).

**Results:**

Five studies (six cohorts) were included in the final analysis. In the pooled analysis, gasping was not only associated with a significant increase in ROSC (RR, 1.87; 95% CI, 1.64–2.13; I^2^ = 70%), but also a high likelihood of favorable neurological outcomes (RR, 3.79; 95% CI, 1.86–7.73), long-term survival (RR, 3.46; 95% CI, 1.70–7.07), and initial shockable rhythm (RR, 2.25; 95% CI, 2.05–2.48).

**Conclusions:**

Current evidence indicates that gasping can predict short and long outcomes in patients with OHCA.In addition, gasping is associated with a high likelihood of initial shockable rhythm,which may contribute to positive outcomes.

## Introduction

Gasping or agonal respirations are abnormal breathing pattern,which are common in the first few minutes after cardiac arrest (CA) [1, 2]. In the 2015 guidelines for CA, gasping has also been highlighted and it was recommended that Emergency Medical Service (EMS) dispatchers should pay more attention to it [3].

It has been reported that the presence of gasping is associated with an increased return of spontaneous circulation (ROSC) and survival to hospital discharge [4, 5]. A meta-analysis was conducted by Zhao et al. to evaluate the impact of gasping on short term outcome. They found that patients who were gasping were 3.5 times more likely to survive to discharge than those without gasping [four studies, five cohorts, 95% confidence interval (CI) 3.0–4.1; *P* < 0.01] and that the presence of gasping predicts ROSC [relative risk (RR) 2.2; 95% CI 1.7–2.8; *P* = 0.02] [6]. However, in this study, the result of ROSC was obtained from a single study, and they did not report the impact of gasping on long term prognosis. After this meta-analysis, several studies regarding gasping in patients with OHCA were published. Wolfskeil et al. founded that a higher gasping rate and deeper negative pressures, but not the presence of gasping, were associated with ROSC [7]. Moreover, there were no meta-analysis conducted to determine whether gasping is associated with an initial shockable rhythm, which can affect CA outcomes. Therefore, to provide the latest and most convincing evidence, we systematically reviewed the current available literature to investigate whether gasping was associated with ROSC. The secondary outcomes were to evaluate whether gasping could predict (1) favorable neurological outcomes at discharge or at 30 days after CA and (2) long-term survival (≥6 months), and (3) whether gasping is associated with an initial shockable rhythm.

## Materials and methods

### Literature search and selection criteria

PubMed, Embase, and Cochrane Library were used to search for records reporting the prognostic effect of gasping on ROSC and long term outcomes in patients with CA. No language restriction was imposed. The last search was run on July 30, 2018. Two independent investigators carried out the initial search, deleted duplicate records, screened the titles and abstracts for relevance, and identified articles that were excluded or required further assessment. The reference lists of relevant studies were also screened in order to identify further studies of interest.

The following search strategy was used for MEDLINE, and was modified to suit other databases.

1. Cardiac arrest
2. CA
3. Cardiopulmonary resuscitation
4. CPR
5. or/1–4
6. Gasping
7. Agonal breathing
8. Agonal respiration
9. or/6–8
10. 5 and 9

Studies meeting the following criteria were included: (1) population: adult patients with OHCA; (2) intervention: gasping; (3) comparison: no gasping; (4) outcome: ROSC, favorable neurological outcome at discharge or at 30 days, long term survival (≥6 months), rate of initial shockable rhythm; and (5) design:observational studies (prospective or retrospective cohort studies).

### Data extraction and quality assessment

Data extraction was performed by Z.Q and confirmed independently by L. B. The following information was extracted from each study: first author, year of publication, country, study design, patient characteristics, number of patients enrolled, intervention, and outcome data. When the same patients were reported in several publications, we retained only the largest study to avoid duplication of information. Discrepancies were resolved by discussion with third the investigators (CS.L). Two investigators (Q.Z and ZJ.Q) independently utilized the Newcastle-Ottawa Scale (NOS) [8] to assess the risk of bias for observational studies.

### Statistical analysis

RevMan 5.3 (Nordic Cochrane Centre) was used to perform the statistical analyses. Differences were expressed as RR with 95% CI. The fixed-effect model were usd when heterogeneity was not present (PQ ≥ 0.1 or I^2^ ≤ 50%), otherwise random-effect model was used (PQ < 0.1 or I^2^ > 50%) [9]. Publication bias was assessed by visually inspecting a funnel plot [10]. The sensitivity analyses were performed only for ROSC due to the limited number of studies for other outcomes. In the sensitivity analyses for observational studies, the influence of a single study on the overall pooled estimate was evaluated by omitting one study in each step.

## Results

### Study identification and selection

**Fig. 1:**
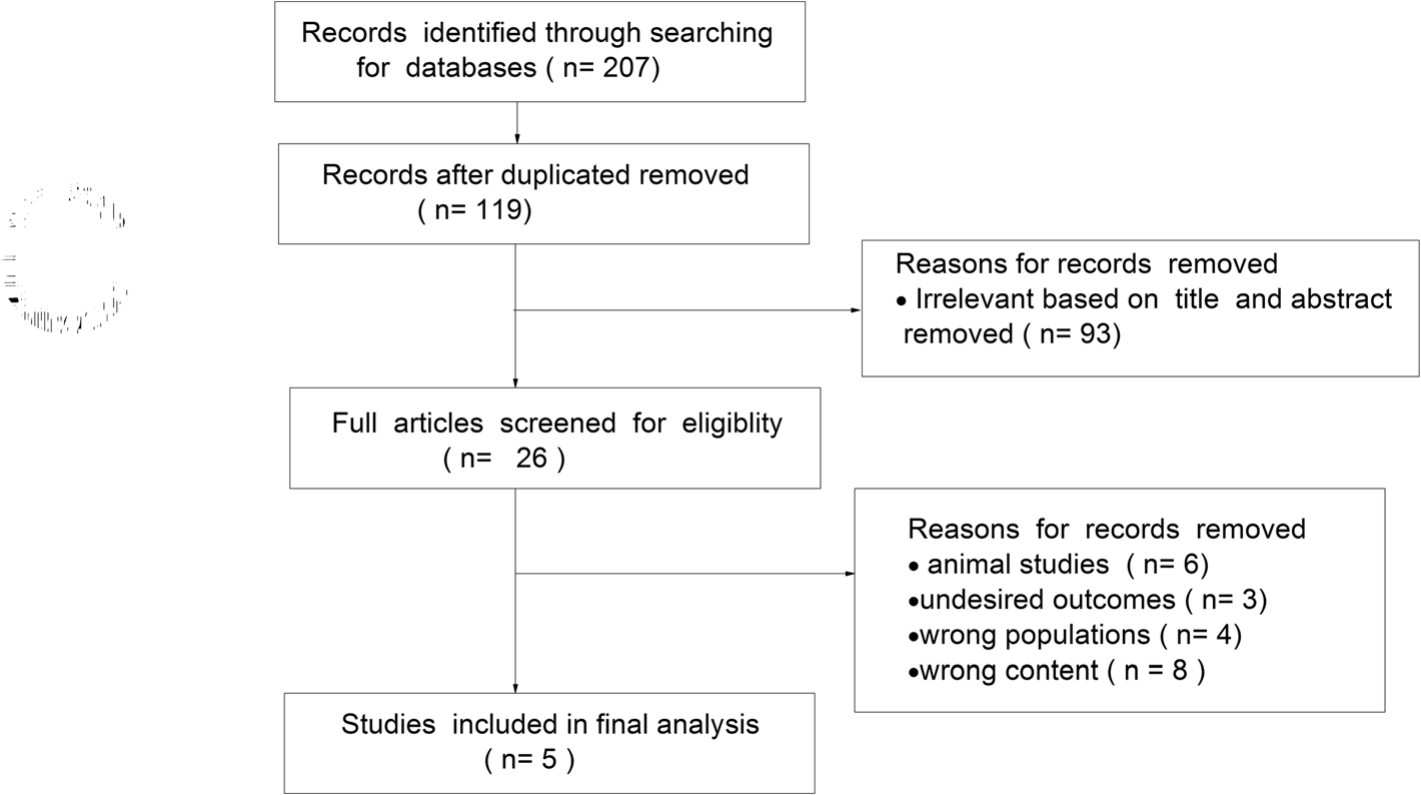
Flow-chart of study selection

A total of 207 records were identified from the initial database search. We excluded 88 duplicate records and 93 records based on the titles and abstracts. The remaining 26 full-text articles were assessed for eligibility, and 21 were excluded for various reasons (i.e., animal studies or different study content or populations). Finally, 5 studies [7, 11–14] were included in the meta-analysis. The selection process is shown in Fig. 1.

### Study characteristics

The main characteristics of the included studies are shown in Table 1. All included studies were observational studies. Among the included studies, there were 2 prospective and 3 retrospective studies. All studies were published in English. Martens et al. reported two databases [12]. All five studies (six cohorts) reported the result of ROSC, two studies [13, 14] reported favorable neurological outcome at discharge or at 30 days after CA, two studies [11, 14] reported long term survival, four studies [7, 11, 13, 14] reported initial shockable rhythm.

**Table1:** Characteristics of included studies

Author	Year	Country	Study Design	Age	Location	Reason of CA	Bystander	Male	Setting	Gasping Time	Number of participants
Martens [12]	1995	Belgium	Retrospective	Adult	OHCA	All	–	71.7%	Single center	EMS	4757
Debaty [14]	2017	France	Prospective	Adult	OHCA	All	42.43%	62.92%	Multi center	EMS	1835
Wolfskeil [7]	2017	Belgium	Prospective	Adult	OHCA	All	–	–	Single center	During Resuscitation	290
Knor [11]	2018	Czece	Retrospective	Adult	OHCA	Cardiac	72.94%	69.02%	Single center	EMS	569
Fukushima [13]	2017	Japan	Retrospective	Adult	OHCA	All	55.2%	65.1%	Multi center	EMS	2411

### Quality assessment

To assess the risk of bias of the cohort studies based on the NOS, 5 studies were rated as a total score of > 7, indicating a low risk of bias (Table 2).

**Table 2:** Risk of bias assessment of the observational studies

Study	Selection				Comparability	Outcome			Total Score
	Exposed Cohort	Nonexposed Cohort	Ascertainment of Exposure	Outcome of Interest		Assessment of Outcome	Length of Follow-up	Adequacy of Follow-up	
Martens	..	*	*	*	* *	*	*	*	8
Debaty	*	*	*	*	* *	*	*	*	9
Wolfskeil	*	*	*	*	*	*	*	*	8
Knor	*	*	*	*	* *	*	*	..	9
Fukushima	*	*	..	*	* *	*	*	*	8

Risk of bias was assessed using the Newcastle-Ottawa Scale. A higher overall score corresponds to a lower risk of bias; a score of ≤5 (out of 9) indicates a high risk of bias

### The primary outcome: ROSC

**Fig. 2:**
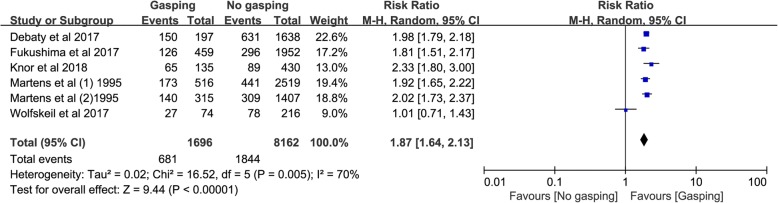
Association of gasping and ROSC in patients with OHCA. df = degrees of freedom; M-H = Mantel-Haenszel

The pooled results from the random-effects model combing the RRs for ROSC is shown in Fig. 2. Overall, 9822 patients were included in this analysis (1696 and 8126 in the gasping and control groups, respectively). Gasping was associated with a significant increase of ROSC (RR, 1.87; 95% CI, 1.64–2.13) with moderate heterogeneity among the studies (I^2^ = 70%; *P* = 0.005).

### The secondary outcomes

**Fig. 3:**
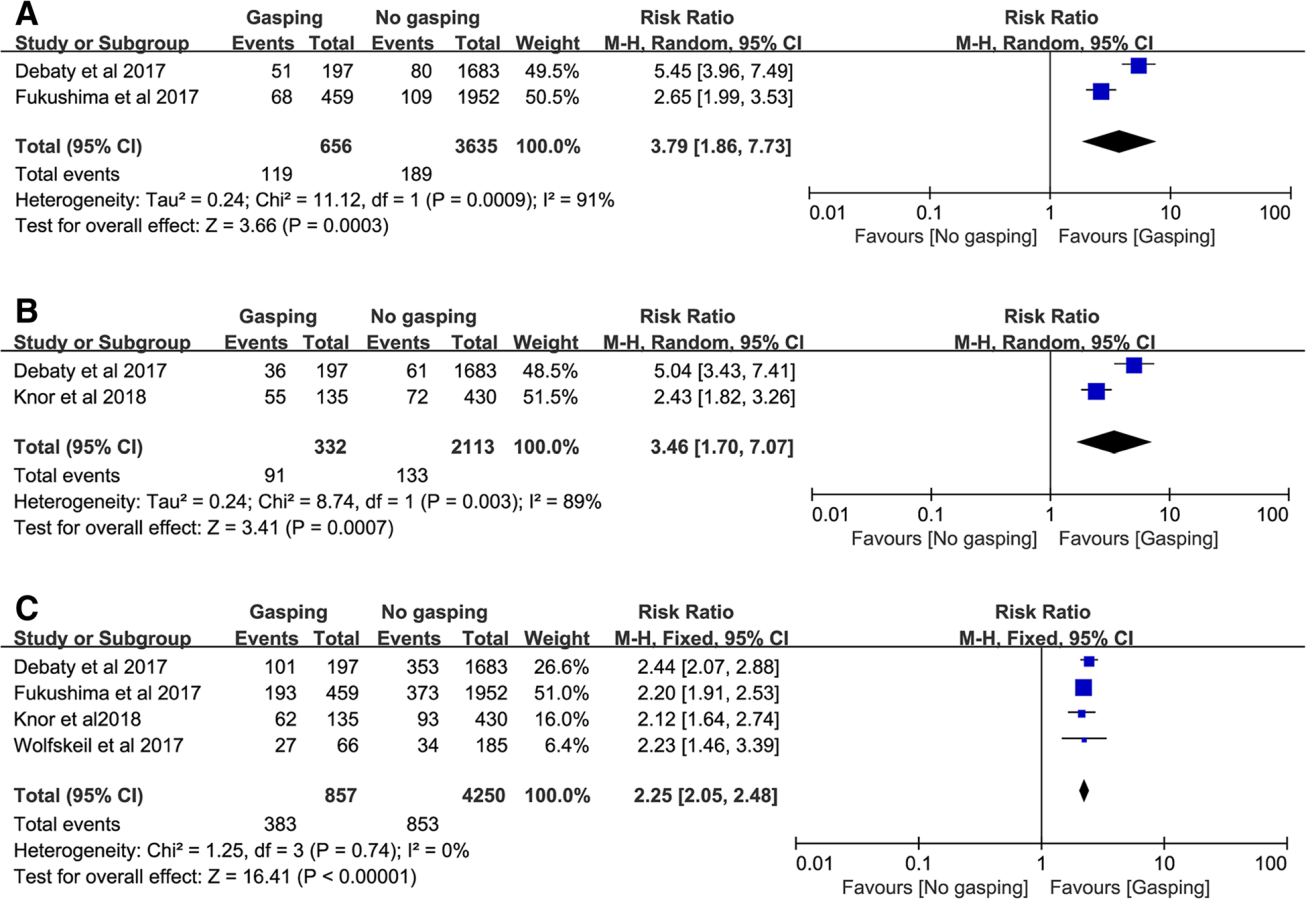
**a** Association of gasping and favorable neurological outcome in patients with OHCA; **b** Association of gasping and long term survival in patients with OHCA; **c** Association of gasping and initial shockable rhythm in patients with OHCA

The secondary outcomes are outlined in Fig. 3. Gasping was associated with an increase of favorable neurological outcomes (RR, 3.79; 95% CI, 1.86–7.73; Fig. 3a), long term survival (RR, 3.46; 95% CI, 1.70–7.07; Fig. 3b), and initial shockable rhythm (RR, 2.25; 95% CI, 2.05–2.48; Fig. 3c).

### Publication Bias

**Fig. 4:**
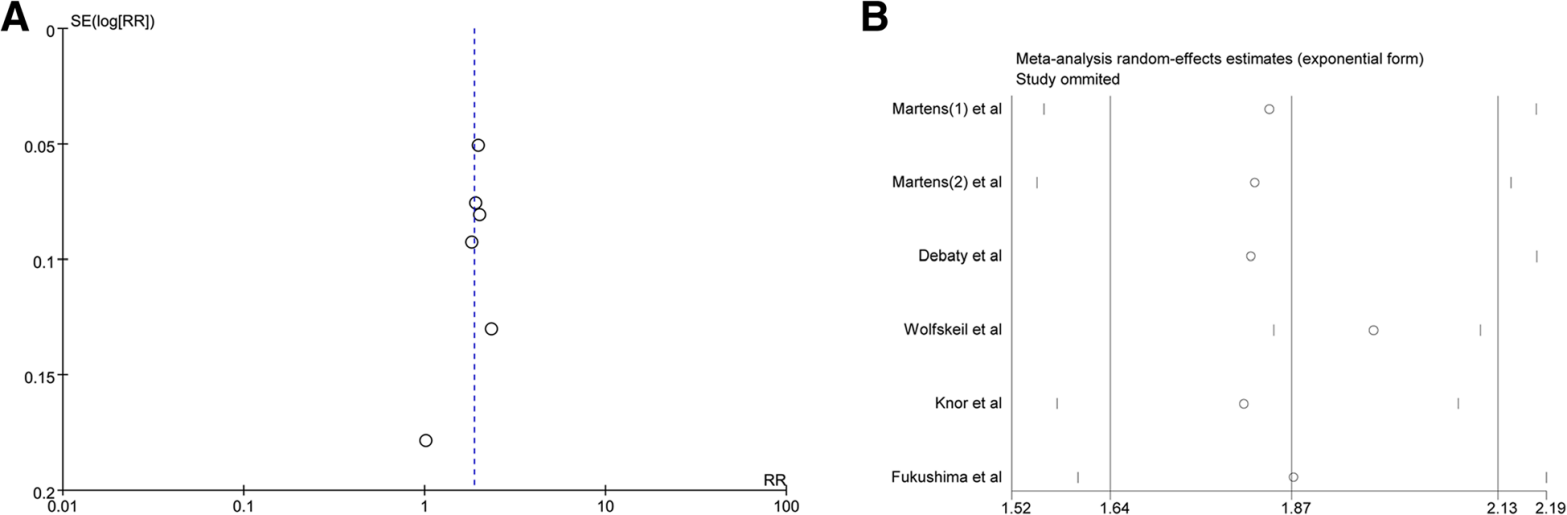
**a** Funnel for ROSC in patients with OHCA; **b** Sensitivity for ROSC in patients with OHCA

For the meta-analysis of gasping on ROSC, there was no evidence of significant publication bias based on the inspection of the funnel plot (Fig. 4a).

### Sensitivity analysis

To further confirm the robustness of the results, we conducted a sensitivity analysis of ROSC, but no significant changes were observed in the outcomes (Fig. 4b).

The results and evidence of heterogeneity were maintained after excluding the studies by Debaty et al. (RR, 1.82; 95% CI, 1.52–2.18; I^2^ = 75%), Fukushima et al. (RR, 1.87; 95% CI, 1.60–2.19; I^2^ = 75%), Knorr et al. (RR, 1.81; 95% CI, 1.57–2.08; I^2^ = 72%), and Martens et al. cohort 1 (RR, 1.84; 95% CI, 1.56–2.18; I^2^ = 76%) and cohort 2 (RR, 1.82, 95% CI, 1.55–2.14; I^2^ = 75%). There was also no change in the pooled results after excluding the study by Wolfskeil et al. (RR, 1.97; 95% CI, 1.85–2.10), but no evidence of heterogeneity was observed among the remaining studies (I^2^ = 0%).

## Discussion

In the present systematic review and meta-analysis, we identified 5 observational studies that were conducted to investigate the prognostic effect of gasping on ROSC and long-term survival in patients with CA. Based on our study analysis, gasping was associated with increased ROSC, which is consistent with previous meta-analysis. In addition, gasping could predict favorable neurological outcomes and long-term survival in patients with OHCA. A high initial shockable rhythm in gasping patients may be one reason for the favorable outcomes.

Gasping is an abnormal breathing pattern which has been defined as an abrupt, sudden and transient inspiratory effort [11]. It is further characterized by short inspiration and expiration and a longer and variable expiratory pause [5].Continued breathing is common during the first minute of CA induced by ventricular fibrillation (VF) [4, 15]. In a pig study of breathing activity after the untreated VF, the airflow pattern was similar to that recorded during spontaneous breathing in the first minute; gasping occurred in a crescendo–decrescendo pattern during the second to fifth minute, and all ventilation activity ceased after the sixth minute of untreated VF [16].

OHCA is a leading cause of death and remains a significant public health problem [17]. The reported percentage of gasping ranges from 33 to 60% in all OHCA patients [18, 19]. Many animal models have been used to study the physiological mechanisms of gasping. Previous researchers have found that gasping could decrease of intracranial pressure, increase of cerebral perfusion, increase flow of ventilation [16, 20, 21]. The beneficial effect of gasping on circulation and gas exchange during CA has been described [22, 23]. Gasping after CA has been linked to the level of brainstem pO2, arterial baroreceptor and chemoreceptor stimulation following a sudden decrease in blood pressure, and arterial acid–base balance [14, 24]. Furthermore, initial shockable rhythms in OHCA were correlation with higher survival rates [25]. Since gasping is present only during the first minutes after onset of CA, the non-gasping group might have simply experienced longer ischemia. This assumption is supported by the finding that gasping is more often associated with initial shockable rhythms,which may contribute favorable outcomes.

Although gasping has beneficial effects for CA patients, immediate recognition of gasping is difficult for the bystander [26]. This can prevent or delay recognition of CA. Riou et al. demonstrated that there is potential for improved recognition of agonal breathing if call-takers are trained [27]. Education and training on caller descriptors could turn these obstacles into opportunities to identify OHCA more quickly and comprehensively [13].

For the present meta-analysis, in an attempt to produce robust results, we pre-stated rigorous inclusion criteria and included only studies that clearly stated the outcomes of OHCA patients. Using the previous meta-analysis as a base, we included four other recent studies to assess effects of gasping on ROSC. With the added statistical power of having 9822 cases as opposed to 5066 cases, our meta-analysis suggested that gasping was associated with increased ROSC. Moreover, the sensitivity analysis and exclusion of any single study did not significantly alter the pooled results, which adds robustness to our main finding. In addition, gasping may predict favorable neurological outcomes and long term survival based on our meta-analysis.

In the present study, we observed moderate or high heterogeneity for the short and long outcome results, which may be due to the different methods for determining the presence of gasping during CA in these 5 studies.Martens et al. [12] and Knor et al. [11] retrospectively reviewed CPR registries, which leaves their data prone to lacking or incorrect entries. Moreover, the study by Martens et al. [12] included cases from more than 30 years ago. Debaty et al. [14] prospectively collected data during a randomized controlled trial comparing standard CPR to active compression/decompression CPR. Wolfkeil et al. [7] did not demonstrate any association between gasping and ROSC. However, that was a special study, where gasping rate, volume and pressure were measured using a dedicated device. As a consequence: gasping was measured only after endotracheal intubation, therefore late on a relatively small patient population and that study may have detected subclinical gasping. Fukushima et al. [13] reviewed audio recordings of emergency callers.

The following points can be considered in further studies. Firstly, there is a need for further education to identify gasping. Secondly, early CPR is crucial for positive CA outcomes, and the use of endotracheal intubation remains debatable in gasping patients. Next, clinical endpoints other than the incidence of gasping, such as gasping volume and gasping rate, should be considered in further studies.

Several potential limitations should be taken into consideration in the present results. Firstly, this finding largely relies on data from observational studies and is potentially subject to selection bias; hence, high-quality and adequately powered studies are warranted. Secondly, other factors that could influence the results, including population characteristics, severity of illness, and reasons for CA, were not considered in the present study. Thirdly, a larger sample size was required to assess the impact of gasping on favorable neurological outcomes and long-term survival. Finally, gasping assessment is a challenging task for rescuer, and the ability to detect gasping can vary among rescuers. Therefore, OHCA patients can be incorrectly assessed as breathing normally.

## Conclusion

Based on the current evidence, gasping could predict short and long outcomes in patients with OHCA. In addition, gasping is associated with a high likelihood of initial shockable rhythm, which may contribute to favorable outcomes.
